# To use indwelling drainage or not in dual-plane breast augmentation mammoplasty patients: A comparative study

**DOI:** 10.1097/MD.0000000000021305

**Published:** 2020-07-17

**Authors:** Yiding Xiao, Jianqiang Hu, Mingzi Zhang, Wenchao Zhang, Feng Qin, Ang Zeng, Xiaojun Wang, Zhifei Liu, Lin Zhu, Nanze Yu, Loubin Si, Fei Long, Yu Ding

**Affiliations:** a Department of Plastic Surgery, Peking Union Medical College Hospital, Beijing; b Department of Orthopaedics, Qingdao Huangdao District Hospital of Traditional Chinese Medicine, Qingdao, Shandong; c Department of Information Engineering, Chaoshan Polytechnic College, Puning, Guangdong, China.

**Keywords:** BREAST-Q scales, drainage, dual-plane breast augmentation mammoplasty

## Abstract

To explore the necessity of indwelling drainage in dual-plane breast augmentation mammoplasty patients.

Female patients (123 in total) were selected from June 2015 to June 2018 in the Department of Plastic Surgery at Peking Union Medical College Hospital and were randomly divided into 2 different groups: the with drainage group (WD group, 57 patients) and the without drainage group (WOD group, 66 patients). In the 2 groups, the operation time, postoperative stay, and hospitalization expenses were recorded. The BREAST-Q Version 2.0 Augmentation Module Pre- and Postoperative Scales (Chinese Version) were used to evaluate psychosocial well-being, sexual well-being, physical well-being, and satisfaction with breasts preoperatively and postoperatively (1 year after operation).

Before the operation, no significant differences were found in psychosocial well-being, sexual well-being, physical well-being, or satisfaction with breasts between these 2 groups. In the WOD group, postoperative stay and hospitalization expenses were remarkably decreased, but the operation time was similar, compared with the WD group. Compared with before the operation, both groups had significantly increased scores in psychosocial well-being, sexual well-being, and satisfaction with breasts after the operation. However, no significant differences were found between the 2 groups. No complications were found in any of the patients.

Although the operation time was not significantly decreased, patients without drainage could save much more time and money and simultaneously reach similar postoperative effects in psychosocial well-being, sexual well-being, physical well-being, and satisfaction with breasts. Therefore, drainage may not be necessary in patients who undergo dual-plane breast augmentation mammoplasty.

## 1 Introduction

Traditionally, there were 2 planes to implant breast prosthesis: the retromammary space and subpectoral space. In 2001, Tebbetts introduced dual-plane breast augmentation, which proved to be a promising and effective approach to offer increased benefits and fewer tradeoffs compared with a single pocket location.^[1]^ Usually, wound drainage is used after plastic and reconstructive surgery, especially augmentation mammoplasty, to reduce potential complications. However, there is limited evidence that shows that drainage truly necessary in breast augmentation. Therefore, a comparative study was established to investigate the necessity of indwelling drainage in dual-plane breast augmentation patients.

## 2 Methods

### 2.1 Patients information

In this study, 123 female patients were selected in the Department of Plastic Surgery at Peking Union Medical College Hospital from June 2015 to June 2018. All patients underwent prosthetic augmentation mammoplasty, and those with breast tumor diseases and history of other breast surgery were excluded. The age of the patients ranged from 18 to 60 years (average age: 36.6341 ± 12.7320 years), the height ranged from 147 to 179 cm (average height: 159.8618 ± 8.1254 cm), the weight ranged from 34 to 75 kg (average weight: 51.1382 ± 7.9324 kg), and the BMI ranged from 15 to 25 kg/cm^2^ (average BMI: 19.9971 ± 2.6012 kg/cm^2^). This clinical study protocol was reviewed and approved by the Bioethical Committee of Peking Union Medical College Hospital in Peking, China. All methods were performed according to the relevant guidelines and regulations. Written informed consent was given by all patients.

### 2.2 Patients grouping

**Figure 1:**
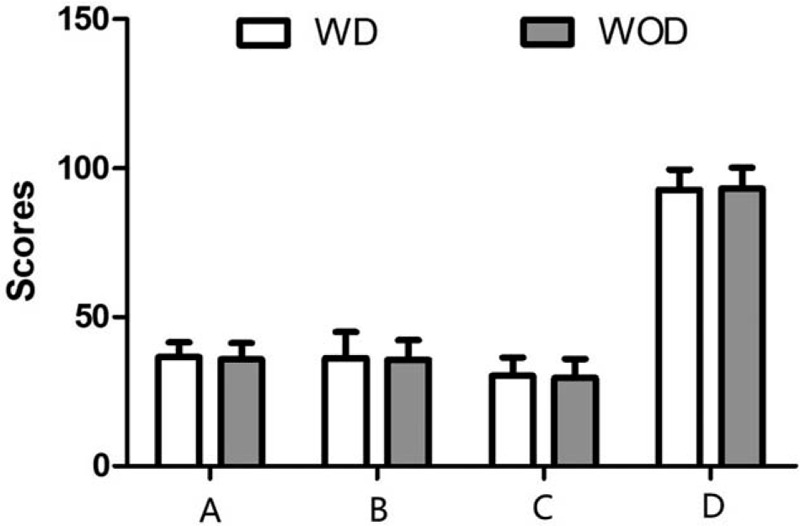
Evaluation of psychosocial well-being (A), sexual well-being (B), satisfaction with breasts (C), and physical well-being (D) through BREAST-Q scales in the WD group and WOD group before the operation. No significant difference was found between 2 groups. Values shown as the mean ± SD (n = 57 in WD group, n = 66 in WOD group). WD = with drainage, WOD = without drainage.

All patients were randomly divided into 2 different groups: the with drainage group (WD group, 57 patients) and the without drainage group (WOD group, 66 patients). Patients in these 2 groups had similar average ages, heights, weights, and BMIs (Table 1). In addition, no significant differences were found preoperatively in psychosocial well-being, sexual well-being, physical well-being, and satisfaction with breasts. (Table 2 and Fig. 1).

**Table 1:**
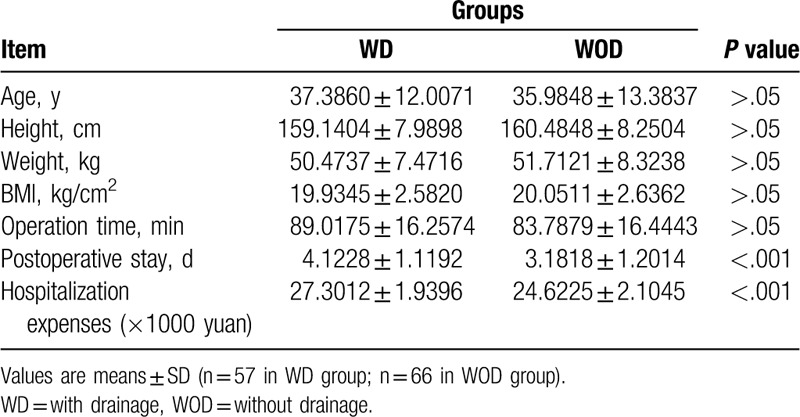
Basic information in WD and WOD groups.

**Table 2:**
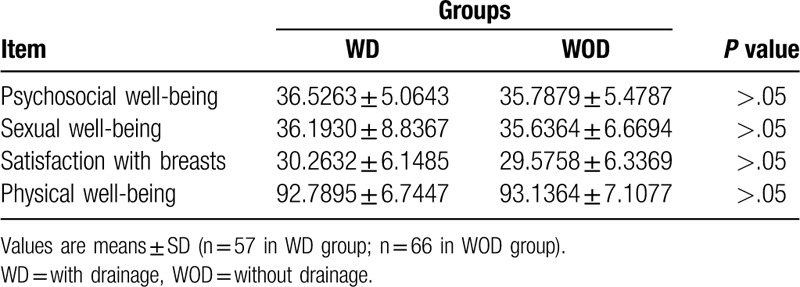
Basic information (evaluated by BREAST-Q Version 2.06 preoperatively) in WD and WOD groups.

### 2.3 Operation procedures and nursing methods

Before the operation, the proper breast prosthesis was chosen according to the breast measurements of patients, and the stripping range was marked on the chests of the patients. A periareolar incision (28 patients in the WD group and 33 patients in the WOD group) and submammary fold incision (27 patients in the WD group and 33 patients in the WOD group) were chosen. All patients received general anesthesia with endotracheal intubation and dual-plane breast augmentation mammoplasty. All procedures were performed visually, and hemorrhage possibilities received preintervention. In the WD group, the drainage tube was set around the submammary fold and pierced the axillary skin to hide the drainage scar. Then, the wound was closed, and the breasts were compressed and fixed properly by a chest belt. In the WOD group, wound closure and breast compression and fixation were followed after implantation with no drainage. Patients in WD group could not leave the hospital until achieving stable vital signs after drainage removal, while patients in the WOD group could not leave the hospital until achieving stable vital signs after the operation. All patients were instructed to wear an elastic bra for 6 months.

### 2.4 Evaluation method

After patients left the hospital, the operation time, postoperative stay, and hospitalization expenses of each patient were recorded. Before the operation and 1 year after the operation, psychosocial well-being, sexual well-being, physical well-being, and satisfaction with breasts were measured by the BREAST-Q Version 2.0 Augmentation Module Pre- and Postoperative Scales Chinese (CN) Version.^[2]^ In addition, complications were recorded.

### 2.5 Statistical analysis

SPSS statistics 24.0 software (SPSS, Inc., Chicago, IL) was used for statistical analysis. Data are presented as the mean ± standard deviation (mean ± SD). The paired samples *t* test was used for scores analysis of 1 single group before and after the operation. The independent samples *t* test was used for other statistical analysis between the 2 groups. A *P* value of <.05 was considered as statistically significant.

## 3 Results

### 3.1 Drainage increased patient postoperative stay and hospitalization expenses

**Figure 2:**
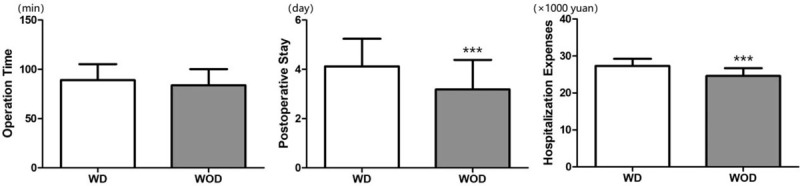
Comparisons of operation time (left), postoperative stay (middle), and hospitalization expenses (right) between the WD group and WOD group. In the WOD group, patients had much shorter postoperative stay and reduced hospitalization expenses compared with the WD group. Operation time showed no significant difference between the 2 groups. Values shown as the mean ± SD (n = 57 in WD group, n = 66 in WOD group; ^∗∗∗^*P* < 0.001). WD = with drainage, WOD = without drainage.

No significant difference was found in the operation time between the WD group and WOD group. Compared with the WD group, the postoperative stay and hospitalization expenses were remarkably decreased in the WOD group (*P* < .001) (Table 1 and Fig. 2).

### 3.2 Operation with or without drainage could increase patients’ psychosocial well-being, sexual well-being, and satisfaction scores

**Figure 3:**
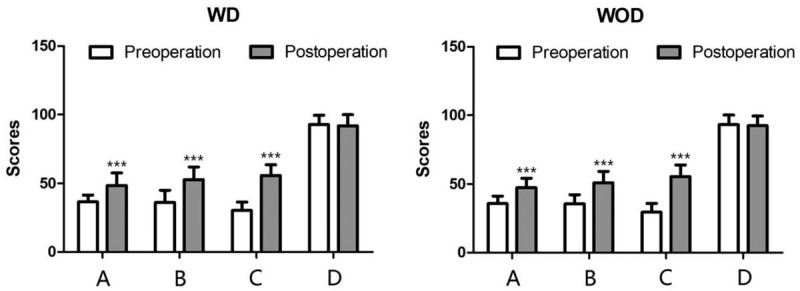
Evaluation of psychosocial well-being (A), sexual well-being (B), satisfaction with breasts (C), and physical well-being (D) through BREAST-Q scales in the WD group (left) and WOD group (right) before and after the operation. In addition to the aspect of physical well-being, the other 3 aspects showed much better amelioration in both the WD group and WOD group postoperatively. Values shown as the mean ± SD (n = 57 in WD group, n = 66 in WOD group; ^∗∗∗^*P* < 0.001). WD = with drainage, WOD = without drainage.

Both in the WD group and WOD group, patients’ psychosocial well-being, sexual well-being, physical well-being, and satisfaction with breasts scores were significantly increased after dual-plane augmentation mammoplasty (*P* < .001). However, physical well-being scores showed similar results preoperatively and postoperatively between the 2 groups (Table 3 and Fig. 3).

**Table 3:**
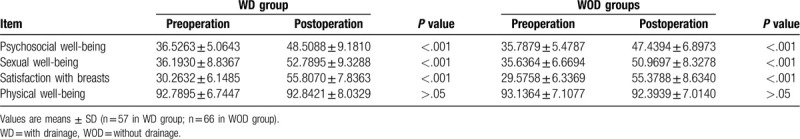
Comparison between preoperative and postoperative items in WD and WOD groups.

### 3.3 Operation with or without drainage may have similar postoperative effects

**Figure 4:**
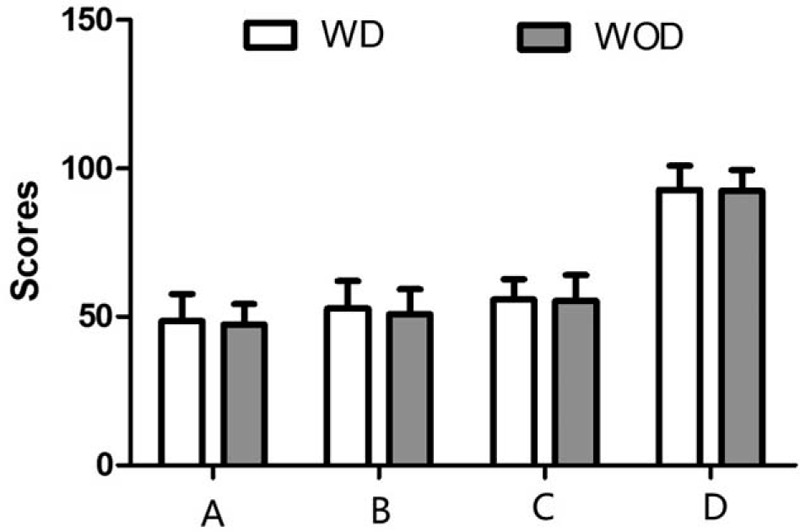
Evaluation of psychosocial well-being (A), sexual well-being (B), satisfaction with breasts (C), and physical well-being (D) through BREAST-Q scales in the WD group and WOD group after the operation. No significant difference was found between the 2 groups. Values shown as the mean ± SD (n = 57 in WD group, n = 66 in WOD group). WD = with drainage, WOD = without drainage.

In the above results, the operation (with or without drainage) increased patients’ psychosocial well-being, sexual well-being, and satisfaction scores. However, the WOD group showed similar results for psychosocial well-being, sexual well-being, physical well-being, and satisfaction with breasts scores compared with the WD group (*P* > .05). (Table 3 and Fig. 4).

Moreover, all patients showed no complications, such as infections, asymmetry or effusion, at 1 year follow-up.

## 4 Discussion

Breast augmentation or augmentation mammoplasty is a popular operation that could modify or achieve satisfying breast appearance. Breast prostheses are common materials in breast augmentation. To implant prostheses, surgeons need to create a proper space. The retromammary space and subpectoral space were traditionally used. In 2001, Tebbetts introduced dual-plane breast augmentation, which is commonly applied in a wide range of breast types.^[1]^

Wound drainage is often used after dual-plane breast augmentation, aiming to reduce potential complications, such as the accumulation of blood and fluid.^[3–5]^ However, other studies have demonstrated that drainage may not ameliorate hematomas or seromas.^[6–8]^ Moreover, vacuum drainage may also cause tube-related tissue injury or hematoma formation,^[9]^ foreign body-related infection,^[10,11]^ blockage,^[12]^ and migration.^[13,14]^ Discomfort and pain may also be associated with a thick drain tube.^[15–17]^ In Khan's study,^[18]^ there was limited evidence available demonstrating no significant benefit of using postoperative wound drainage in reduction mammoplasty. However, no data are available for breast augmentation. This study aims to offer available and reliable data about the necessity of indwelling drainage in dual-plane breast augmentation mammoplasty patients.

In this study, 132 patients were selected and divided into 2 different groups, which shared similar basic patient information and BREAST-Q scores of psychosocial well-being, sexual well-being, physical well-being, and satisfaction with breasts. Obviously, the operation with or without drainage increased BREAST-Q scores (besides physical well-being). However, no significant differences were found between the WD group and WOD group after dual-plane breast augmentation mammoplasty, which indicated that indwelling drainage may not be an important factor to achieve satisfying postoperative effects. Applying no drainage could save nearly 6 minutes during the operation, but it is still insignificant. It is well accepted that hospital stay and costs could be increased by using drainage,^[19–21]^ which was also concluded in this study.

In recent years, increasing numbers of studies have questioned the necessity of indwelling drainage,^[7,20,22,23]^ and these studies have demonstrated similar or better clinical effect in postoperative patients. In this study, the results in the WOD group showed similar postoperative appearance and physical–psychological satisfaction with shorter hospital stay and less cost, which indicated that drainage may not be essential in patients who undergo dual-plane breast augmentation mammoplasty.

In addition, plastic surgeons need to pay much more attention to the following 3 points when deciding to not use indwelling drainage. Not using indwelling drainage could be considered when a periareolar incision and submammary fold incision are used. In this case, the surgeon could perform the operation visually to avoid creating excessive space. Preintervention for all visible hemorrhage possibilities is better than stopping bleeding afterwards. Proper compression and fixation are more important early after the operation.

## 5 Conclusion

Drainage may not be essential in patients who undergo dual-plane breast augmentation mammoplasty.

## Author contributions

**Study administration:** Ang Zeng.

**Data collection:** Yiding Xiao, Jianqiang Hu, Wenchao Zhang, Feng Qin.

**Data analysis:** Yiding Xiao, Mingzi Zhang, Xiaojun Wang, Zhifei Liu, Lin Zhu.

**Figures and tables:** Nanze Yu, Loubin Si, Fei Long, Yu Ding.

**Writing:** Yiding Xiao, Mingzi Zhang, Ang Zeng.
